# Surgical Cases

**Published:** 1878-09

**Authors:** Charles A. Wall


*ART. III.—Surgical Cases* in the private practice of Prof. J. F. Miner, M. D. Reported by Charles A. Wall, B. S.

Case V. Mrs. J. was married when over thirty, and had one child, since which time she has been an invalid. Many and varied had been the opinions given to her by the various physicians whom she consulted, until at length, upon a close examination by the aid of a speculum, a laceration of the cervix was discovered, and on July 29th, 1877, after she was brought under the influence of ether, the uterus was drawn down by long tenaculum forceps, and the sides of the laceration pared. The uterus was then allowed to ascend to its normal position. The parts healed readily, all discharge soon ceased and she expressed herself as feeling better than for years. This lesion of the os uteri was formerly overlooked, and only of late has the attention of the profession been called to their occasional occurence, even now they are ignored by many. In most cases careful examination of the neck of the uterus is absolutely necessary to a correct diagnosis, for the injury is seemingly so small and incapable of causing so much trouble, but when met with it will explain many blind symptoms otherwise obscure. Relaxing the tension upon the organ, it was observed that there was a strong tendency to return to normal condition, the pared surfaces closing upon each other so perfectly that no sutures appeared necessary, and none were used.

Case VI. Mrs. S., about forty years of age, had vesico-vaginal fistula caused by a long tedious labor with application of forceps. To the latter, as is usual in such cases, she attributed her condition. The fistula was midway between the external parts and the os uteri, and easily reached and would admit a small catheter. May 30, 1878, after insensibility had been induced by the use of ether the fistula was pared, laterally removing all cicatricial tissue, and the edges approximated by five silver sutures. The operation was successful, and in two weeks she was dismissed, perfectly recovered.

Case VII. Mrs. C., American, married, has never born children, and for over a year past has complained of uterine trouble. Upon examination an unpromising condition was found. The os was softened, dilated and unhealthy, and frequent and profuse hemorrhage occurred. The os having been widely dilated by large sponge tents. July, 1878, she was brought under the influence of an anæsthetic, and two fibroid tumors removed. They were easily enucleated, the uterus having been pulled down to view. They were situated in the lower portion of the cervix, which was much enlarged and granular; the uterus was normal in size and position. The removal of the local lesion caused all other symptoms to subside, and she soon recovered her wonted health. The case is unusual in that, the tumors, although they were each about the size of walnuts, did not cause the usual sympathetic enlargement of the uterus, disturbances of the nervous system, or the lumbar pains, so often complained of in such conditions. The case presented but one symptom of the presence of sub-mucous uterine fibroid.

Case VIII. Mrs. Emma F., American, aged 32, had noticed four years ago a slight tumor in the left inguinal region, upon severe pressure. It was only within a year that it had enlarged enough to become troublesome, and in the last three months it had doubled in size, until she was immense; the antero-posterior diameter just below the umbilicus, being over two feet, and the circumference nearly five; when she sat in a chair the abdomen extended beyond the knees, and was supported on the thighs. Her weight was one hundred and fifty-two; her height being about five feet and two inches; the bones of her arms and legs seemed to be covered with integument as thin as paper, every elevation or depression distinctly marked. So emaciated was she, that little hope of any surgical assistance could be offered; however she strongly insisted, willing to try any chance that an operation offered, so that it was at last granted, October 9th, 1877, in the presence of Drs. Bailey, Dayton, Brush and W. W. Miner, and three medical students. The preliminary incision and evacuation of the contents of the cyst were done as commonly, but the tumor was found to be very large, having an extremely short pedicle and closely adherent to nearly every organ in the abdomen. It was impossible to remove the tumor entire, and so it was carefully enucleated from its attachment, and in those places where this could not be done, a portion of the cyst was cut out, leaving it as a portion of living tissue, receiving its blood supply from its attachments. No large vessels were met with, but some of the smaller could be traced 12 or 15 inches before they entered the cyst walls. These were peeled off and allowed to fall into the abdomen and not trimmed from fear of hemorhage, which was consequently the minimum. The right ovary was also diseased and similarly removed. During the operation the patient's pulse was weak, and she did not rally well; but after brandy had been given she brightened up and appeared cheerful. 4, P. M., six hours after operation, pulse 100; temperature normal, intellect clear, and although she did not exhibit any other symptoms, died from exhaustion upon the sixth day. There were no indications of peritonitis, nor of pyemia, but she had not strength to sustain the depression attending the removal of so large a tumor, whose weight was over ninety-five pounds. The result was as anticipated, but she was gratified by the trial of the only means having the possibility of a favorable issue. Attention is called to the foregoing case, that where large adhesions which cannot be separated without danger of hemorrhage are met with, the sac may be cut around its attachments, and the portion remaining will be no cause of danger. It is living tissue, not a foreign body, and can do no possible harm. There is less danger in allowing even large pieces of the sac to remain, than in forcing it from its strong and firm attachment.

